# The construction of an *Agrobacterium*-mediated transformation system of *Gynostemma pentaphyllum* using the phosphomannose-isomerase/mannose selection system

**DOI:** 10.5511/plantbiotechnology.23.0418a

**Published:** 2023-06-25

**Authors:** Muxiu Tan, Fengming Liu, Yueying Xie, Qiaocheng Mo, Fenghua Shi

**Affiliations:** 1 Guangxi Botanical Garden of Medicinal Plants, 189 Changgang Road, Nanning 530023, Guangxi Zhuang Autonomous Region, China

**Keywords:** *Agrobacterium tumefaciens*, *Gynostemma pentaphyllum*, PMI, transformation system

## Abstract

In this study, the transformed system mediated by *Agrobacterium tumefaciens* of *Gynostemma pentaphyllum* was constructed by using the phosphomannose-isomerase (PMI) gene as a marker. To investigate the cefotaxime sodium salt (Cef) concentration of bacteriostatic medium and the appropriate mannose concentration in the selectable medium, explants of the stems with buds were cultured in a basic medium supplemented with different Cef and mannose concentrations, respectively. After these were optimized, 288 explants were transformed according the protocol described above to verify their availability by using the polymerase chain reaction (PCR), reverse transcription-PCR and chlorophenol red. The results showed that the appropriate Cef concentration for bacteriostatic culture and mannose concentration for selectable culture were 150 mg l^−1^ and 3 g l^−1^ for stem with buds, respectively. According to the PCR results, the transformation frequency of stems with buds was 20.49% with a regeneration rate of 29.16%. In future, the CPR assay could be the auxiliary method of choice as it is moderately accurate, but it has good maneuverability and is cost effective for large-scale use.

## Introduction

*Gynostemma pentaphyllum* (Thunb.) (*G. pentaphyllum*) is a perennial herb belonging to the cucurbitaceae family, and it has many pharmacological activities, such as anti-inflammatory, anti-oxidant, anti-tumor, anti-platelet clotting and anti-thrombotic properties. It is also able to regulate lipid metabolism, hypoglycemia, enhance immunity, anxiety symptoms and it can protect the liver. It is used to treat hyperlipaemia, oral diseases, non-alcoholic fatty liver disease and tumors (Shen et al. 2020). The *G. pentaphyllum* species is wide spread throughout Asia and there are 21 subspecies. It is widely distributed in southwestern China.

As a plant which is homologous with medicine and food, *G. pentaphyllum* has a long history of use. At first, it was used as an edible wild herb. Later, its dried leaves were used as tea and its effects listed above were found (Yuan et al. 2019). Since panaxadiol saponin was first isolated from *G. pentaphyllum* in 1976 (Nagai et al. 1976), approximately 100 dammarane-type saponin glycosides have been found, and among these there were 8 ginsenosides (Lou et al. 2021). The discovery of dammarane-type saponin glycosides made *G. pentaphyllum* a prominent candidate for use in traditional Chinese medicines because of the well-known biological activity of ginsenosides. Continuous cropping obstacles and the long production cycle associated with *Panax ginseng* (Choi 2008), have resulted in the high production cost of ginsenosides. *G. pentaphyllum* could be a useful and a low cost alternative substitute for *P. ginseng* in order to produce ginsenosides as it has strong environmental adaptability and a short production cycle. In order to transform *G. pentaphyllum* into abiological reactor of ginsenoside, a transformation system was necessary for the introduction of an exogenous gene.

The hairy root cultures of *G. pentaphyllum* are mediated by *Agrobacterium rhizogenes* which has previously been constructed (Chang et al. 2005), but a transformation system mediated by *Agrobacterium tumefaciens* has not be reported until now. Transformation systems have been constructed for many other medicinal plants. An appropriate selection marker has to be found in order to establish an efficient transformation system. The phosphomannose-isomerase (PMI) gene, encoding the PMI enzyme, has been used extensively as a selection marker in many plants (Bříza et al. 2008; Degenhardt et al. 2006; Feeney and Punja 2003; Lucca et al. 2001; O’Kennedy et al. 2004; Ramesh et al. 2006; Jain et al. 2007; Joersbo et al. 1998; Wang et al. 2013; Wright et al. 2001; Zhang and Puonti-Kaerlas 2000; Zhang et al. 2015). Commercialized genetically engineered maize (Herman et al. 2021) and high vitamin-A rice were also bred though transformation systems using the PMI gene as the selectable marker, and the latter has received regulatory food, feed, and/or cultivation approval in Australia, Canada, New Zealand, Philippines and the United States (Su et al. 2020). Compared with traditional antibiotics and herbicides systems, the obvious advantages of the PMI/mannose selectable system lie in its higher transformation efficiency. More importantly, PMI is deemed to be safe to human and animal health as well as the environment as the gene is widespread in nature. In addition, maize expressing the PMI protein had been grown widely with no known reports of adverse health effects including allergic responses (Allen et al. 2018).

In this study, the PMI gene was used to distinguish the transformed from the untransformed cells. Transgenic detection showed that stems with buds of *G. pentaphyllum* were transformed successfully with a transformation frequency of 20.69%. Here we describe the protocol of the transformation program which uses the PMI/mannose selection system.

## Materials and methods

### Determination of the optimum cefotaxime sodium salt (Cef) concentration for bacteriostatic culture

Stems with buds at lengths of 0.8–1.0 cm from the sterile rooting plants were inoculated into the basic medium supplemented with 0, 50, 100, 150 and 200 mg l^−1^ Cef. Every treatment had 3 replicates and 6 bottles were used per replication with 8 explants per bottle. Traits regarding the calluses and buds were recorded at 30 days after inoculation.

### Determination of mannose concentration for selection culture

The stems with buds were sliced into 0.8–1.0 cm from the proliferation shoots, and these were inoculated into the induction medium supplemented with various concentrations of mannose in combination with sucrose. The following mixtures were used during the first round: 0 g l^−1^+28 g l^−1^, 4 g l^−1^+24 g l^−1^, 8 g l^−1^+20 g l^−1^, 12 g l^−1^+16 g l^−1^, 16 g l^−1^+12 g l^−1^, 20 g l^−1^+8 g l^−1^, 24 g l^−1^+4 g l^−1^ and 28 g l^−1^+0 g l^−1^. In the second round, the following were used: 0 g l^−1^+28 g l^−1^, 1 g l^−1^+27 g l^−1^, 2 g l^−1^+26 g l^−1^, 3 g l^−1^+25 g l^−1^ and 4 g l^−1^+24 g l^−1^. The traits regarding the calluses and buds were recorded at 30 days after inoculation.

### Plasmid and transformation

Professor Guibing Hu of the Horticultural College South China Agriculture University provided the plasmid, pCAMBIA1301-PMI. This was transformed into *Agrobacterium tumefaciens* LBA4404 by using anelectroporator. *Agrobacterium tumefaciens* strain LBA4404, containing pCAMBIA1301-PMI, was cultured at 28°C in YEP liquid medium with Kan (50 mg l^−1^) and Rif (100 mg l^−1^) at 220 revolutions/minute (rpm). When the OD value reached 0.8–1.0, the bacteria suspension was centrifuged at 4,000 rpm for 5 min. The pellets at the bottom of tubes were re-suspended in the liquid induction medium, and cultured at 28°C at 220 rpm until its OD value reached 0.4–0.6. The bacterial suspension was kept at 4°C until needed.

After pre-culture for 2–3 days, the explants were immersed in bacteria medium for 10 min. These were successively co-cultured under darkness for 2–4 days and were kept as bacteriostatic culture for 5 days. Finally, they were transferred to the selection medium. All explants were grown at 25°C with 12 h light per day. All the different media were used at pH 5.8–6.2 and are listed in Table 1.

**Table 1:** Table 1. The composition of the medium at every cultural stage for transformation.

Cultural stage	Medium
Bud induction	MS +2.0 mg l^−1^ 6-BA +0.02 mg l^−1^ NAA +5.5 g l^−1^ agar +28 g l^−1^ sucrose
Pre-culture	MS +2.0 mg l^−1^ 6-BA +0.02 mg l^−1^ NAA +5.5 g l^−1^ agar +28 g l^−1^ sucrose
Co-culture	MS +2.0 mg l^−1^ 6-BA +0.02 mg l^−1^ NAA +5.5 g l^−1^ agar +28 g l^−1^ sucrose
Bacteriostatic culture	MS +2.0 mg l^−1^ 6-BA +0.02 mg l^−1^ NAA +5.5 g l^−1^ agar +28 g l^−1^ sucrose +150 mg l^−1^ Cef
Determined selection culture	MS +2.0 mg l^−1^ 6-BA +0.02 mg l^−1^ NAA +5.5 g l^−1^ agar +25 g l^−1^ sucrose +3.0 g l^−1^ mannose +150 mg l^−1^ Cef
Root induction	1/2 MS +0.2 mg l^−1^ IAA +5.5 g l^−1^ agar +28 g l^−1^ sucrose +150 mg l^−1^ Cef

### PCR determination and PMI activity assay

Buds with the 3–4 leaves in the selectable medium were assessed by using PCR, RT-PCR and PMI activity assays. Genomic DNA was extracted from leaves of the transformed buds by cetyltrimethyl ammonium bromide. 40 ng DNA was used as template in a 25 µl reaction mixture with the primer pair, PMI1F: 5′-CACTGCGTGATGTGATTGAGAG-3′ and PMI1R: 5′-CGGCTGGAGTAGGGAGACA-3′. The program for PCR was conducted with an initial denaturing step at 95°C for 5 min, followed by 30 cycles of 30 s denature at 95°C, 30 s of primer-annealing at 55°C and 1 min of synthesis at 72°C. Then there was a final extension at 72°C for 5 min. Total RNA was extracted from the leaves of the transformed buds by TRIzol reagent (Invitrogen), and then reversed-transcribed by using a PrimeScript RT reagent Kit with gDNA Eraser (Takara). 1 µl of transcribed cDNA was added to the 25 µl reaction system with the same primer pair and the program above was used to conduct the PCR. All PCR products were resolved by using 1.7% agarose gels.

PMI activity was measured by using the chlorophenol red (CPR) assay. Infected leaves of buds were sliced and surface-sterilized with 70% ethanol for 30 s and this was followed by 0.1% HgCl_2_ for 5 min. They were then rinsed in distilled water three times and transferred to liquid medium with 1/2 MS medium (pH 6.4) supplemented with 2.5 g l^−1^ of mannose and 50 mg l^−1^ of CPR dye. The color of the CPR was assessed after incubation at room temperature for 24 h in darkness.

### The effects of rooting and transplanting on positively transformed seedlings

When the height of positive seedlings reached about 3 cm, they were transferred into the rooting medium which was composed of 1/2 MS +5.5 g l^−1^ agar +28 g l^−1^ sucrose +150 mg l^−1^ Cef +0.2 mg l^−1^ IAA. The data regarding the root traits were collected one month after inoculation into the rooting medium.

The good quality positive plantlets were selected for acclimatization. They were refined for 5–7 days in a culture flask with a closed lid, and further refined for another 2–3 days with an opened lid at room temperature. During the refining period, the plantlets were sprayed 4–5 times with water. After refining, the plantlets were taken out from the culture flask and transplanted into nutrition bowl containing a matrix of nutritional soil and vermiculite at a volume ratio of 1 : 1. The environmental humidity was kept above 70% during cultivation. The growth situation of these plantlets were investigated and their survival rates were calculated one month after being transplanted.

### Statistical analysis

Data from all experiments were subjected to ANOVA. The means were compared using SPSS software (version 20.0) and by carrying out the Duncan’s multiple range test.

## Results

### Determination of Cef concentration in the induction medium

In order to obtain the appropriate concentration of Cef to restrain the *agrobacterium*, the stems with buds were inoculated in the bud induction medium with different concentrations of Cef. The survival rates of explants and the callus rate were recorded. The texture, color and quality of the callus were observed, as well as the bud rate and the number of buds per explants, bud height, bud color and quality after 30 days of inoculation. The results are shown in Table 2. It was found that the Cef concentration had little effect on the survival rate of explants and traits regarding the calluses. However, there were obvious effects on the number of buds per explants and the bud height. Other traits such as the bud induction rate, the bud color and quality were also not affected by the Cef concentration. Furthermore, the higher the concentration of Cef, the more the buds per explants and the higher the buds obtained. When the Cef concentration was 150 mg l^−1^, the number of buds obtained was at its maximum and when this rose to 200 mg l^−1^, the buds grew the fastest.

**Table 2:** Table 2. The effects of different Cef concentrations on the induction of buds.

Cef concentration (mg l^−1^)	Bud induction rate (%)	Bud number of explants	Height of bud (cm)	Color of bud	Quality of bud
0	100.00±0.00a	3.59±0.23b	2.78±0.16b	green	+++
50	100.00±0.00a	4.01±0.33ab	2.70±0.15b	green	+++
100	93.33±0.83b	4.16±0.39ab	2.85±0.07b	green	+++
150	100.00±0.00a	5.00±0.67a	2.77±0.16b	green	+++
200	100.00±0.00a	4.66±0.48ab	3.58±0.26a	green	+++

Each value represents the mean±SE of three replicates, each with 48 explants. Data in the same column followed by different letters are significantly different at *p*≤0.05 level, according to Duncan’s multiple range test.

### Determination of mannose concentration in the selection medium

**Figure 1:**
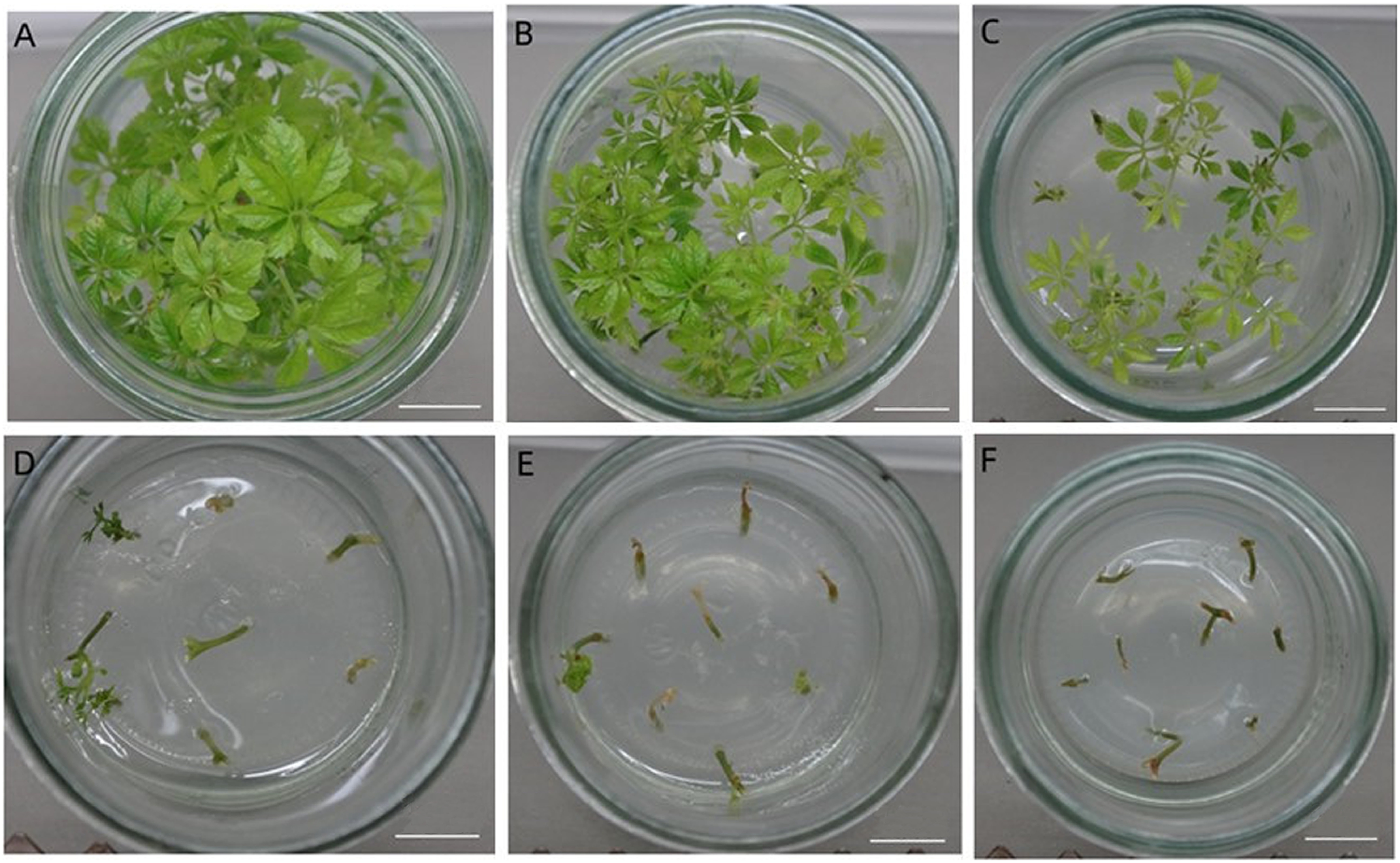
Figure 1. The effects of different concentrations of mannose and sucrose on bud induction after 30 days of inoculation. A: 0 g l^−1^ mannose +28 g l^−1^ sucrose, B: 1 g l^−1^ mannose +27 g l^−1^ sucrose, C: 2 g l^−1^ mannose +26 g l^−1^ sucrose, D: 3 g l^−1^ mannose +25 g l^−1^ sucrose, E: 4 g l^−1^ mannose +24 g l^−1^ sucrose, F: 8 g l^−1^ mannose +20 g l^−1^ sucrose. Bar (A–F) represents 1 cm.

In order to investigate the tolerance of *G. pentaphyllum* to mannose, stems with buds were inoculated in the bud induction medium supplemented with different concentrations of mannose and sucrose. The results are shown as Tables 3 and 4, and Figure 1. It was found that mannose had a significant effect on the survival rate of explants, callus formation and bud induction of explants. The explant survival rate, callus formation rate and diameter, bud formation rate, bud numbers and height decreased with increasing concentrations of mannose. The bud quality also declined. When the mannose concentration rose from 0 to 4 g l^−1^, the survival rates of explants decreased from 100 to 45.69%, the callus induced rates from 100 to 0% and the bud rate from 99.17 to 23.75%, respectively. The bud number per explants also declined from 4.5 to 1.1 and the bud height decreased from 2.42 to 0.28 cm, respectively. When mannose concentration rose to 16 g l^−1^, no buds were induced. Consequently, 0–4 g l^−1^ of mannose was the appropriate range used for selection. To further decide the appropriate mannose concentration, we inoculated the stems with buds in the induction medium with mannose concentrations from 0–4 g l^−1^. It was found that when the mannose concentration was increased to 3.0 g l^−1^, the bud induction rate was 26.10%. Consequently, 3.0 g l^−1^ of mannose was added into the bud induction medium and this was the appropriate concentration to effect the selection for transformation of *G. pentaphyllum*.

**Table 3:** Table 3. The effects of different mannose concentrations during the first round on the bud induction of explants.

Mannose/sucrose (g l^−1^)	Callus formation rate (%)	Explants survival rate (%)	Callus diameter (cm)	Bud formation rate (%)	Bud number per explants	Bud height (cm)	Bud color	Bud quality
0/28	100	100.00±0.00a	0.23±0.02a	99.17±0.83a	4.50±0.22a	2.42±0.22a	Light-green	++
4/24	0	45.69±9.45b	0.00±0.00b	23.75±4.02b	1.10±0.10b	0.28±0.02b	Light-green	++
8/20	0	23.33±5.07c	0.00±0.00b	3.33±3.00c	0.33±0.33cd	0.08±0.08b	Light-green	+
12/16	0	9.03±1.84d	0.00±0.00b	2.08±1.20c	0.67±0.33bc	0.30±0.15b	Light-green	+
16/12	0	4.28±3.15d	0.00±0.00b	0.00±0.00c	0.00±0.00d	0.00±0.00b	−	−
20/8	0	1.04±1.04d	0.00±0.00b	0.00±0.00c	0.00±0.00d	0.00±0.00b	−	−
24/4	0	0.00±0.00d	0.00±0.00b	0.00±0.00c	0.00±0.00d	0.00±0.00b	−	−
28/0	0	0.00±0.00d	0.00±0.00b	0.00±0.00c	0.00±0.00d	0.00±0.00b	−	−

Each value represents the mean±SE of three replicates, each with 48 explants. Data in the same column followed by different letters are significantly different at *p*≤0.05 level, according to Duncan’s multiple range test.

**Table 4:** Table 4. The effects of different mannose concentrations during the second round on the bud induction of explants.

Mannose/sucrose (g l^−1^)	Callus formation rate (%)	Explants survival rate (%)	Callus diameter (cm)	Bud formation rate (%)	Bud number per explants	Bud height (cm)	Bud color	Bud quality
0/28	100.00±0.00a	100.00±0.00a	0.43±0.03a	100.00±0.00a	5.63±0.59a	2.71±0.27a	green	+++
1/27	100.00±0.00a	100.00±0.00a	0.31±0.01b	100.00±0.00a	5.45±0.39a	2.72±0.10a	green	+++
2/26	61.11±7.35b	100.00±0.00a	0.24±0.01c	86.12±4.22b	2.26±0.11b	1.01±0.10b	green	+++
3/25	0.00±0.00c	99.17±0.83a	0.00±0.00d	26.10±4.62c	1.17±0.17c	0.36±0.05c	green	+
4/24	0.00±0.00c	94.58±2.92b	0.00±0.00d	4.31±1.18d	1.00±0.00c	0.17±0.03c	green	+

Each value represents the mean±SE of three replicates, each with 48 explants. Data in the same column followed by different letters are significantly different at *p*≤0.05 level, according to Duncan’s multiple range test.

### Verification of the transformation system

**Figure 2:**
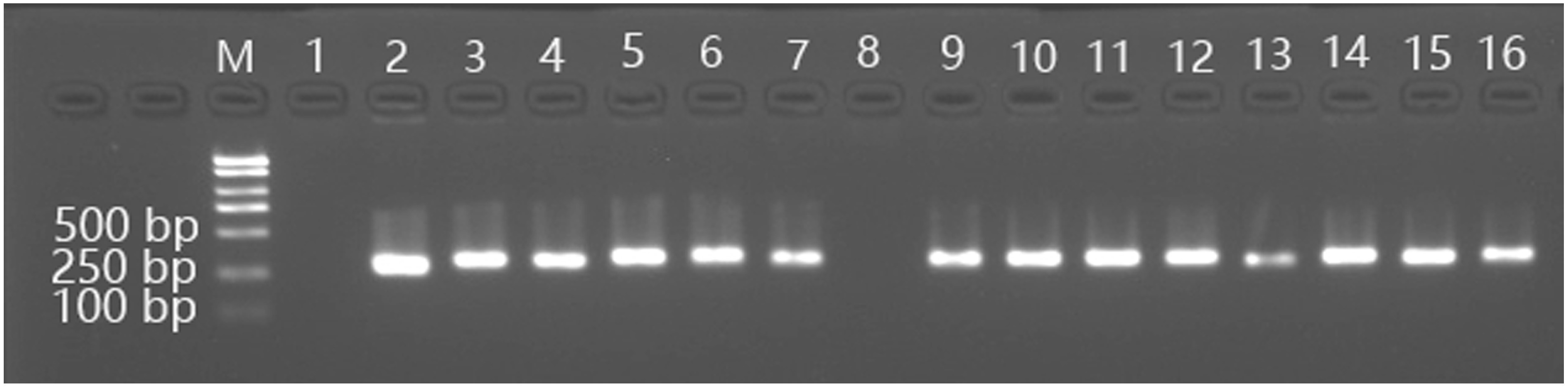
Figure 2. RT-PCR test for the putatively transformed buds of *G. pentaphyllum*. M: DNA marker; 1: untransformed leaves; 2: pCambia1301-PMI plasmid; 3-16: putatively transformed leaves of L3, L5, L6, L11, L21, L29, L32, L33, L44, L58, L59, L72, L76 and L77, respectively.

**Figure 3:**
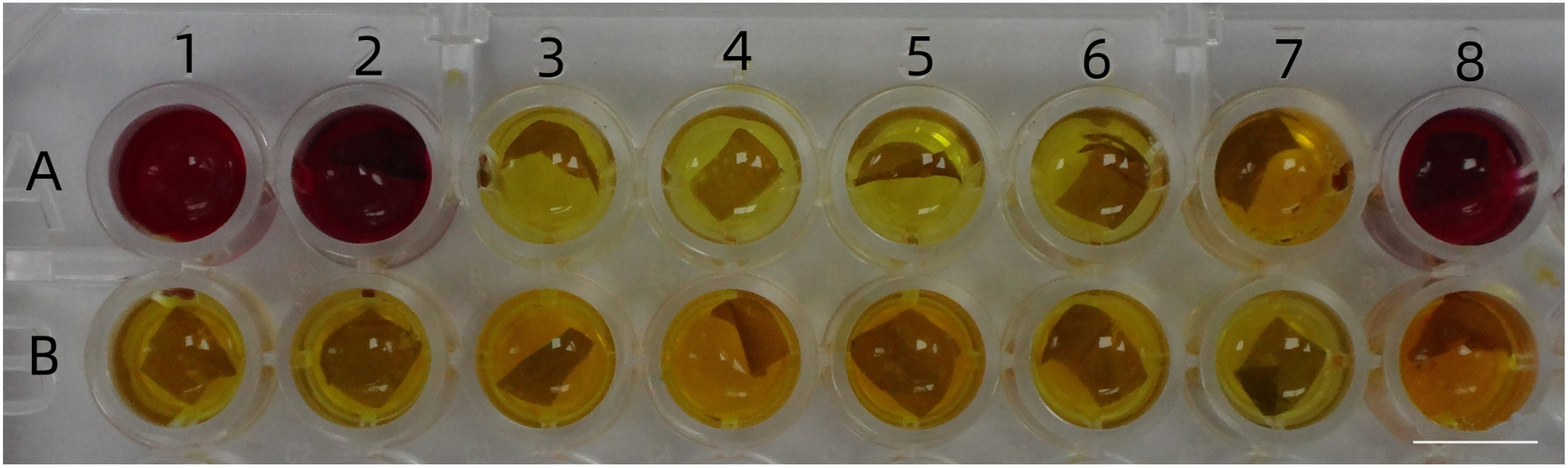
Figure 3. CPR assays for the putatively transformed buds of *G. pentaphyllum*. A1: blank control; A2: untransformed control; A3–A8, B1–B8: putatively transformed leaves of L3, L5, L6, L11, L21, L29, L32, L33, L44, L58, L59, L72, L76 and L77, respectively. Bar represents 0.5 cm.

288 explants of stems with buds were transformed according the above procedure including pre-culture, infection, co-culture and bacteriostatic culture with 150 mg l^−1^ of Cef. Then these were screened in selection medium supplemented with 3.0 g l^−1^ of mannose and 25.0 g l^−1^ of sucrose. 84 plants were subsequently harvested. They were all tested by PCR using a specific primer pair for the PMI gene, and 59 plants were found to be positive. The bud induction rate was 29.16%, and the transformation frequency was 20.49%. The results of transformed samples using RT-PCR and CPR are showed in Figures 2 and 3.

PMI encodes the PMI enzyme which can convert mannose-6-phosphate into fructose-6-phosphate. Mannose metabolism decreases the pH of the medium, and in the presence of CPR, this turns red. In order to investigate the corresponding relationship of the results obtained from PMI gene amplification and PMI activity assays, some of the samples were also subjected to the CPR assay. The data obtained are shown in Table 5. The CPR results of 23 samples were different from those of PCR, and 21 of these had no PMI activity, but they were positive according to PCR. This indicated that the CPR assay can sometimes give false negative results for the transformation with respect to the selection of the PMI gene of *G. pentaphyllum*.

**Table 5:** Table 5. Summary of PMI expression measured by PCR and CPR assays in the putatively transformed buds.

Sample No	PCR	CPR	Sample No	PCR	CPR	Sample No	PCR	CPR
1	+	−	30	+	+	66	−	+
2	+	−	31	+	−	67	−	−
3	+	+	32	+	+	68	+	+
4	+	+	33	+	+	69	+	−
5	+	+	34	+	−	70	+	−
6	+	+	42	−	+	71	+	−
7	+	+	44	+	+	72	+	+
10	+	+	45	+	−	73	−	−
11	+	+	46	+	−	74	+	−
12	+	+	49	+	+	75	+	−
13	+	+	50	+	+	76	+	+
14	+	−	51	+	+	77	+	+
21	+	+	52	+	+	78	+	−
22	+	−	53	−	−	79	+	+
23	+	−	55	−	−	80	+	−
24	+	−	58	+	+	81	+	−
26	+	+	59	+	+	82	+	−
28	+	+	64	+	+	83	+	−
29	−	−	65	−	−	84	+	−

+: The test result was positive, −: The test result was negative.

### The effects of rooting and transplanting on positively transformed seedlings

**Figure 4:**
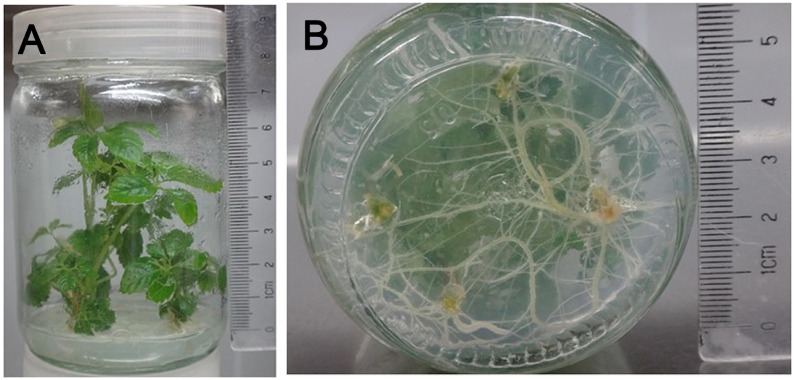
Figure 4. The effects of rooting culture observed on the seedlings after one month. A: The overall effects rooted seedlings, B: The roots of rooted seedlings.

**Figure 5:**
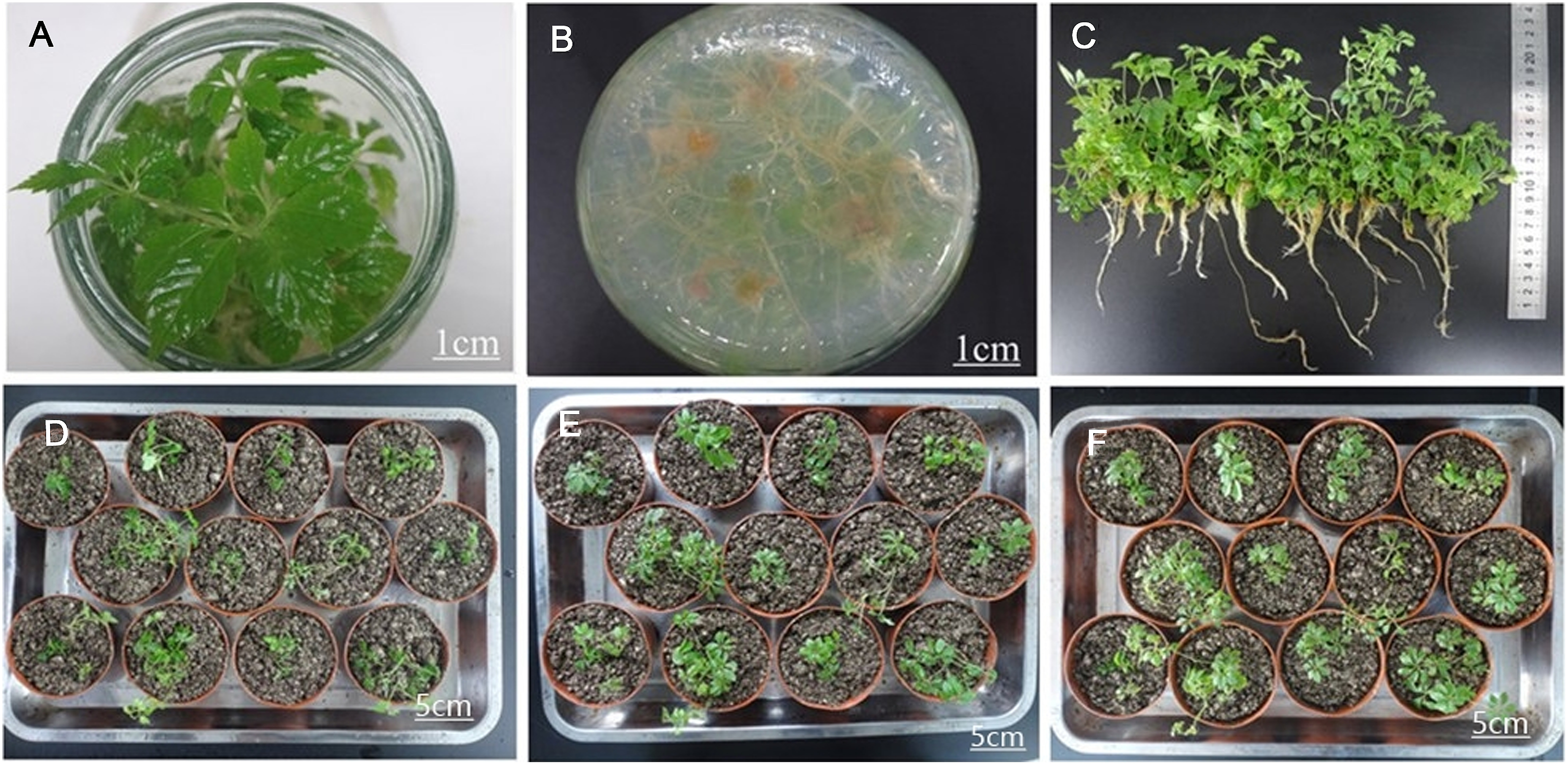
Figure 5. The characteristics of rooted culture seedlings after transplantation. A: The branches and leaves during acclimatization, B: The roots during acclimatization, C: The whole plantlets after complete washing of the rooting media during acclimatization, D: Day 0 after transplantation, E: Day 3 after transplantation, F: Day 14 after transplantation. Bar (A, B) represents 1 cm. Bar (D–F) represents 5 cm.

These plantlets grew strong, with a height of approximately 6.08 cm. Their root color changed from beige to light green and the average rooting rate and root numbers were 100% and 17.01 respectively. The effect of root induction is shown in Figure 4. The acclimatized plantlets grew strongly for one month after being transplanted. At that time their survival rate was 91.67%, and their lengths and diameters of the main bines were 15 and 0.10–0.15 cm, respectively. Their overall observed characteristics are shown in Figure 5.

## Discussion

As for most plants, the PMI gene does not exist in *G. pentaphyllum*, and 16 g l^−1^ of mannose in its growth medium could completely suppressed bud induction. Thus, the PMI/mannose selection system could be used during the transformation of *G. pentaphyllum*. Its transformation frequency was 20.49%, which was higher than rice cultivar “93-11” (7.5%) (Hou et al. 2021), *L. hypoglauca* (15.69%) (Shi et al. 2020) and *Prunus domestica* (1.40%) (Sidorova et al. 2017). It was lower than several other plants including apple (24%) (Degenhardt et al. 2006), cucumber (23%) (He et al. 2006), sweet orange (23.8%) (Boscariol et al. 2003), oilseed rape (24.2%) (Wang 2009) and lettuce (25%) (Bříza et al. 2010). In view of the relatively high transformation frequency, it was concluded that the selection system of PMI/mannose was suitable for the transformation of *G. pentaphyllum*.

According to previous studies (Gui et al. 2014; Joersbo et al. 1998), a high transformation frequency can be achieved by increasing the regeneration rate, which could be achieved by reducing the selection pressure. In our study, the bud induction rate was about 26.10% when 3 g l^−1^ mannose was supplemented in the medium. The mannose concentration in selection medium needed to be further decreased when the regeneration rate reached to about 50%. In view of accessibility, the leaves should also be suitable explants. However, the buds were difficult to induce from leaf explants and the highest bud induced rate achieved was 25% (Liu et al. 2012; Zhao 2007). Thus, it is necessary to search for an effective recipe in order to perform bud induction from the leaves.

The CPR assay can distinguish transformed from untransformed cells, and therefore be used it to investigate positive plants. Our study showed us that assessment of positivity by PCR was not always same as the results obtained with CPR. False negative results were predominant when using the CPR assay, which was also found in other plants such as crowtoe (Guo et al. 2015) and *L. hypoglauca* (Shi et al. 2020). This maybe due to either to gene splicing resulting from the insertion occurring in improper locations or that excessive copies were formed. Different degrees of color intensity were also found in our study, which may have resulted from the copy number obtained and the location where the gene was inserted (Allen et al. 2018; Gui et al. 2014; Guo et al. 2015). If we assume the PCR results to be correct, then the accuracy of the CPR assay was 73.84%, which was higher than that observed for *L. hypoglauca* at 67.74% (Lucca et al. 2001). This method could also be used as an auxiliary method in order to identity the positive plants in the standardized protocol.

In this study, it was found that budding rate as well as the bud color and quality were little affected by a range of Cef concentrations (50–200 mg l^−1^) when these were supplemented in the bud induction medium. Conversely, the number of bud per explants and the bud height increased as the Cef concentration was increased. This phenomenon maybe due to the presence of endophytic fungi and bacteria (Shang et al. 2021; Zhang et al. 2015), which were associated with contamination of the explants. We also observed that the plants which grew in the medium containing Cef were robust when compared to those that grew in its absence. However, this study showed *G. pentaphyllum* readily accepted an exogenous gene, and this plant may prove to be a promising substitute for ginseng in its ability to produce ginsenosides.

## Abbreviations

Cef: cefotaxime sodium salt
CPR: chlorophenol red
IAA: 3-indolyl acetic acid
Kan: kanamycin sulfate
NAA: naphthylacetic acid
PCR: polymerase chain reaction
PMI: phosphomannose-isomerase
Rif: rifampicin
RT-PCR: reverse transcription-polymerase chain reaction
6-BA: 6-benzylaminopurine
